# RI2AP: Robust and Interpretable 2D Anomaly Prediction in Assembly Pipelines

**DOI:** 10.3390/s24103244

**Published:** 2024-05-20

**Authors:** Chathurangi Shyalika, Kaushik Roy, Renjith Prasad, Fadi El Kalach, Yuxin Zi, Priya Mittal, Vignesh Narayanan, Ramy Harik, Amit Sheth

**Affiliations:** 1 Artificial Intelligence Institute, College of Engineering and Computing, University of South Carolina, Columbia, SC 29208, USA; kaushikr@email.sc.edu (K.R.); kaippilr@mailbox.sc.edu (R.P.); yzi@email.sc.edu (Y.Z.); mittalpriya1321@gmail.com (P.M.); vignar@sc.edu (V.N.); 2 McNair Center for Aerospace Innovation and Research, Department of Mechanical Engineering, College of Engineering and Computing, University of South Carolina, Columbia, SC 29201, USA; elkalach@email.sc.edu (F.E.K.); harik@mailbox.sc.edu (R.H.)

**Keywords:** anomaly prediction, smart manufacturing, assembly processes, sensor data, time series analysis

## Abstract

Predicting anomalies in manufacturing assembly lines is crucial for reducing time and labor costs and improving processes. For instance, in rocket assembly, premature part failures can lead to significant financial losses and labor inefficiencies. With the abundance of sensor data in the Industry 4.0 era, machine learning (ML) offers potential for early anomaly detection. However, current ML methods for anomaly prediction have limitations, with F1 measure scores of only 50% and 66% for prediction and detection, respectively. This is due to challenges like the rarity of anomalous events, scarcity of high-fidelity simulation data (actual data are expensive), and the complex relationships between anomalies not easily captured using traditional ML approaches. Specifically, these challenges relate to two dimensions of anomaly prediction: predicting when anomalies will occur and understanding the dependencies between them. This paper introduces a new method called Robust and Interpretable 2D Anomaly Prediction (RI2AP) designed to address both dimensions effectively. RI2AP is demonstrated on a rocket assembly simulation, showing up to a 30-point improvement in F1 measure compared to current ML methods. This highlights its potential to enhance automated anomaly prediction in manufacturing. Additionally, RI2AP includes a novel interpretation mechanism inspired by a causal-influence framework, providing domain experts with valuable insights into sensor readings and their impact on predictions. Finally, the RI2AP model was deployed in a real manufacturing setting for assembling rocket parts. Results and insights from this deployment demonstrate the promise of RI2AP for anomaly prediction in manufacturing assembly pipelines.

## 1. Introduction

The manufacturing industry has witnessed multiple evolutionary iterations throughout its history. From the mechanization of Industry 1.0, the mass production of Industry 2.0, the automation of Industry 3.0, and, finally, today’s era of smart manufacturing of Industry 4.0 [1]. Each of these revolutions is characterized by specific capabilities introduced to manufacturing systems to evolve these systems. The era of Industry 4.0 has transformed the manufacturing landscape with the advent of data-driven smart manufacturing, a paradigm aiming at utilizing generated data to influence decision-making processes to improve productivity and efficiency [2].

Time series data have become ever-present within manufacturing systems with the proliferation of affordable and robust sensors available in the market. Hence, time series analytics have experienced significant progress in Industry 4.0. An estimated one trillion sensors are projected to be utilized in manufacturing facilities by 2025 [3]. The time series sensor data involved in manufacturing processes can play a pivotal role in analytics-driven insights into events of interest, such as anomalies.

Specifically, we are interested in utilizing the time series data to predict future anomalies based on historical data and the current status of the manufacturing system [4,5]. However, being able to accurately predict anomalous events in production lines can be challenging. Real manufacturing datasets can be very imbalanced, as it is rare for anomalies to occur in mature manufacturing processes [6]. Translating the data into meaningful insights about anomalies (e.g., remedial actions) can be challenging due to the considerable number of sensors that must be considered. Lastly, the interdependence between the sensor data and anomaly categories further complicates the prediction problem.

To tackle these challenges, researchers have experimented with data-driven statistical learning- and ML-based solutions for anomaly prediction. The spectrum of methods explored includes traditional statistical approaches like ARIMA, exponential smoothing, and structural models, as well as ML and neural network methods such as gradient boosting, convolutional neural networks, recurrent neural networks, and their variations [7,8,9,10,11,12,13,14,15]. More details on these early works are available in Section 2 and Appendix A. In recent times, researchers have drawn inspiration from the success of generative artificial intelligence (GenAI). This has led to exploring pre-trained foundational time series models such as TimeGPT and PromptCast. These models are fine-tuned for specific downstream tasks, such as anomaly prediction [16,17].

Although the methods explored so far have shown promise, they have not achieved adequate predictive performances (the SOTA F1 measure is 50% in prediction and 66% in detection— Appendix C) due to several key challenges that still remain: (i) a robust solution for modeling the *rarity* of anomalous occurrences, e.g., rocket parts being fitted poorly, do not frequently occur in mature assembly pipelines, often resulting in a poor predictive accuracy; (ii) a framework for modeling the *two-dimensional* nature of the problem, namely, the prediction of the anomaly(s) at future time steps, along with dependencies among the anomalies when more than one occurs; and (iii) a lack of high-fidelity simulation data corresponding to real-world rocket assembly pipelines (the data generated often lack the stochasticity of real-world pipelines). Beyond prediction-related challenges, there are also hurdles related to interpreting the result in a domain-expert-friendly manner for informing insights into improving pipelines [18].

*We propose a novel framework for handling the abovementioned challenges, which we refer to as Robust and Interpretable 2D Anomaly Prediction (RI2AP). Our main contributions are as follows:*

**Figure 1:**
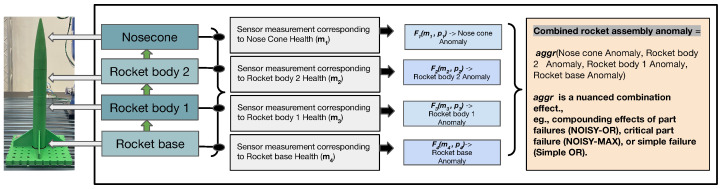
Shows an abstract illustration of the RI2AP method proposed in this work. Sensor measurements correspond to the health of different rocket parts. Several function approximations are then used to predict anomalous occurrences from the sensor measurements, and their outputs are combined using combining rules. The combining rules allow natural aggregation mechanisms, e.g., NOISY-OR and NOISY-MAX, as shown in the illustration.

- For challenges (i) and (ii) above, we implemented the following strategies. We model an anomaly using a *compositional real-valued number*. First, we encode each anomaly class using a monotonically increasing token assignment strategy (e.g., 0 for none, 1 for the first part falling off, 2 for the second part falling off, and so on). This is done to capture the monotonically increasing nature of the severity of anomaly categories in rocket assembly. Next, we represent compositional anomalies using the expected value of their token assignments. We propose a novel model architecture that predicts both the sensor values at the next time step, as well as the value assigned to the compositional anomaly (hence the name 2D prediction). The robustness to *rarity* is achieved through modeling the problem using a regression objective, thus preventing the need for obtaining an adequate number of positive vs. negative class instances or other ad hoc sampling strategies to handle the rare occurrence.
- For challenge (iii), we use the Future Factories dataset. The dataset originates from a manufacturing assembly line specifically designed for rocket assembly, adhering to industrial standards in deploying actuators, control mechanisms, and transducers [19].
- For enabling domain-expert-friendly interpretability, we introduce combining rules first introduced in the independence of a causal influence framework [20], which were specifically inspired by real-world use cases such as healthcare cases to allow enhanced expressivity beyond traditional explainable AI (XAI) methods (e.g., saliency and heat maps). We note that although XAI methods are useful for the system developer for debugging and verification, they are not end-user friendly and do not give end-users the information they want [18]. We demonstrate how combining rules allows natural and user-friendly ways for the domain expert to interpret the influence of individual measurements on the prediction outcome.
- This full investigation aimed to tackle the above challenges to create an adequate model and fully deploy this model in a real manufacturing system. The results and insights from this deployment showcase the promising potential of RI2AP for anomaly prediction in manufacturing assembly pipelines.
  Figure 1 shows a summary of the proposed method.

The rest of this paper is organized as follows. Section 2 covers past work on anomaly detection and prediction within manufacturing processes using univariate and multivariate sensor data. Through this literature survey, we identify the key research gaps. Section 3 describes the dataset and summary statistics. Section 4 introduces a precise formulation of the problem aimed at addressing the gaps identified in Section 2. Section 5 details the proposed solution approach (the RI2AP method), design motivations, and other architectural choices (e.g., function approximator choices). Section 6 provides our experimental setup and records the improvements of our proposed approach over state-of-the-art baselines for a robust proof-of-concept (POC) model. Section 7 covers the deployment of the POC model on the Future Factories manufacturing cell. This includes the deployment plan, technical details, deployment results, and issues faced in deployment. We conclude the paper in Section 8 by summarizing the significant this study’s findings and limitations and avenues for future work.

## 2. Related Work

Various studies have been conducted on anomaly detection and prediction within manufacturing processes, specifically leveraging univariate or multivariate sensor data and employing various algorithmic methodologies. These methods can be categorized into four major groups: supervised classification and regression [21,22,23,24,25,26], clustering [27], meta-heuristic optimization [28], and advanced learning [29] methods.

Wang et al. [21] proposed a method based on recurrent neural networks to detect anomalies in a diesel engine assembly process, utilizing routine operation data, reconstructing input data to identify anomaly patterns, and providing insights into the time step of anomaly occurrences to aid in pinpointing system issues. Ref. [22] addressed the problem of unexpected assembly line cessation with a unique approach that integrates Industrial Internet of Things (IIoT) devices, neural networks, and sound analysis to predict anomalies, leading to a smart system deployment that significantly reduces production halts. Ref. [23] investigated and developed automatic anomaly detection methods using support vector machines for in-production manufacturing machines. They considered operational variability and wear conditions, achieving a high recall rate without continuous recalibration, specifically in the rotating bearing of a semiconductor manufacturing machine. Ref. [24] conducted fine-grained monitoring of manufacturing machines, addressing challenges in data feeding and meaningful analysis, analyzing real-world datasets to detect sensor data anomalies in pharma packaging, and predicting unfavorable temperature values in a 3D printing machine environment. They developed a parameterless anomaly detection algorithm based on the random forest algorithm and emphasized the efficiency of anomaly detection in supporting industrial management. The research conducted by Abdallah et al. [25] analyzed sensor data from manufacturing testbeds using deep learning techniques, evaluated forecasting models, demonstrated the benefit of careful training data selection, utilized transfer learning for defect-type classification, released a manufacturing database corpus and codes, and showed the feasibility of predictive failure classification in smart manufacturing systems. Park et al. [26] proposed a fast adaptive anomaly detection model based on an RNN Encoder–Decoder and using machine sounds from Surface-Mounted Device (SMD) assembly machines. They utilized Euclidean distance for abnormality decisions, and the proposed approach has its structural advantages over Autoencoders (AEs) for faster adaptation with reduced parameters.

Chen et al. [27] developed a novel Spectral and Time Autoencoder Learning for Anomaly Detection (STALAD) framework for in-line anomaly detection in semiconductor equipment, utilizing cycle series and spectral transformation from equipment sensory data (ESD). They implemented an unsupervised learning approach with Stacked Autoencoders for anomaly detection, designing dynamic procedure control, and demonstrating its effectiveness in learning without prior engineer knowledge. Saci et al. [28] developed a low-complexity anomaly detection algorithm for industrial steelmaking furnaces using vibration sensor measurements, optimizing parameters with multiobjective genetic algorithms, demonstrating a superior performance over SVM and RF algorithms, and highlighting its suitability for delay-sensitive applications and limited computational resources devices, with a generic applicability to industrial anomaly detection problems. Ref. [29] investigated anomaly detection and failure classification in IoT-based digital agriculture and smart manufacturing, addressing technical challenges such as sparse data and varying sensor capabilities. The study evaluated ARIMA and LSTM models, designed temporal anomaly detection and defect-type classification techniques, explored transfer learning and data augmentation methods, and demonstrated improved accuracies in failure detection and prediction. However, to the best of the authors’ knowledge, none of the studies have studied how to model the interdependencies of anomalies in a manufacturing setting.

## 3. Future Factories Dataset

We used the Future Factories (FF) dataset [30] generated by the Future Factories team operating at the McNair Aerospace Research Center at the University of South Carolina, which has been made available publicly. A visual representation of the FF setup is included in Appendix E. The dataset consists of measurements from a simulation of a rocket assembly pipeline, which adheres to industrial standards in deploying actuators, control mechanisms, and transducers. The data consist of several assembly cycles with several kinds of measurements, such as the conveyor variable frequency, drive temperatures, conveyor workstation statistics, etc., for a total of 41 measurements. In this work, we first utilized, XGBoost 2.0.1, and its coverage measure to narrow down 20 out of the 41 measurements that contain high information content. XGBoost has achieved a SOTA performance on anomaly *detection* and *prediction* (prediction refers to the identification before the anomalous event, and detection refers to the identification after the event), and therefore, we used it to narrow down our feature selection (please refer to Appendix B for coverage plots and an example of a learned tree from the XGBoost model). Each assembly cycle is associated with one among eight different anomaly types. Upon domain expert consultation, we further grouped the anomaly types into five distinct categories: a *None* type, *Type 1:* one rocket part is missing, *Type 2:* two rocket parts are missing, *Type 3:* three rocket parts are missing, and *Type 4:* miscellaneous anomalies. Table 1 and Table 2 describe the dataset and anomaly statistics, respectively.

## 4. Problem Formulation

In this section, we formally characterize the problem. We begin with clarifying notations denoting the dataset components, followed by the encoding method for the target variable, i.e., the anomalous events. Equipped with the appropriate notations, we describe the task that we aimed to solve in this work.

### 4.1. Notations

Consider an assembly cycle that assembles a rocket from the set of parts $P=\{p_{1},p_{2},p_{3},\ldots \}$. Parts $p_{i}$ with lower values for *i* represent parts at the rocket’s lower end; otherwise, higher values for *i* represent parts at the rocket’s upper (or nose) end. Each cycle occurs over a sequence of t=1,2,…,T discrete time steps. We referred to [30] for details on the definition of a time step (e.g., sampling rate). At each time step *t*, a group of 20 sensor measurements are collected (see Section 3); we denote them as the set $M^{t}=\left\{m_{1}^{t},m_{2}^{t},\ldots ,m_{20}^{t}\right\}$. Anomalies during a cycle are recorded by a separate mechanism and categorized as *None* or *Types 1–4* as in Section 3. We denote anomaly *Type 1* as the singleton tuple $a_{1}=\left(p_{i}\right),\;p_{i}\in P$, and *Type 2* as the two-tuple $a_{2}=\left(p_{i},p_{j}\right),\;p_{i},p_{j}\in P,\;i<j$, *Type 3* as the three-tuple $a_{3}=\left(p_{i},p_{j},p_{k}\right),\;p_{i},p_{j},p_{k}\in P,\;i<j<k$. In a single cycle, parts falling off follow a compositional pattern, where the bottom parts of the rocket detach before the top parts. However, the time gap between these occurrences is nearly instantaneous and cannot be captured within discrete time steps. Consequently, only one type of anomaly from the set $A=\{a_{0},a_{1},a_{2},a_{3},a_{4}\}$ is recorded at each time step. It is important to note that, in reality, a combination of failures can occur. This is why we define each anomaly using indexed parts $p_{i},p_{j},p_{k},i<j<k$, where the ordering of the indices is representative of the spatial structure of the rocket (bottom to top). The miscellaneous anomaly type *Type 4* is denoted as $a_{4}=\left(p_{i},p_{j},p_{k}\right),\;p_{i},p_{j},p_{k}\in P$. The ordering of indices is not important since they correspond to crashes (see Table 2) and are, therefore, unrelated to the spatial structure of the rocket. Finally, the *None* type is denoted as $a_{0}=\left(None\right)$. In the next subsection, we describe how the anomalies were encoded in our work given the above notations.

### 4.2. Anomaly Encodings

Recall *A* to be the set $\left\{a_{0},a_{1},a_{2},a_{3},a_{4}\right\}$. To capture the compositional nature of the anomalies, we perform token assignments to each anomaly type as follows: $token\left(a_{0}\right)=\left[0\right]$, $token\left(a_{1}\right)=\left[i\right]$, $token\left(a_{2}\right)=\left[\frac{i+j}{2}\right]$, and $token\left(a_{3}\right)=\left[\frac{i+j+k}{3}\right]$. It is clear that this token assignment is monotonically increasing, which is representative of the spatial structure of the rocket, and also captures an increasing degree of severity (more parts falling off vs. fewer parts falling off, as mentioned in the main contributions from Section 1). For $a_{4}$, we perform the token assignment as $token\left(a_{4}\right)=max\left(\left\{token\left(a_{3}=\left(p_{i},p_{j},p_{k}\right)\right)|p_{i},p_{j},p_{k}\in P\right\}\right)+1$, i.e., miscellaneous anomalies are assigned the maximum possible value since they correspond to crashes that are considered the most severe. Note that anomaly *Type 4* is not related to the spatial structure of the rocket.

### 4.3. Why Not Simple “One-Hot” Encoding for Anomaly Types?

Extensive prior work on anomaly detection for the specific case of rocket assembly studied in this paper has shown that “one-hot” encoding and other similar data reformatting techniques lead to poor performances for ML classifiers. Appendix C shows the SOTA results achieved using “one-hot”-encoded labels. Our problem formulation more naturally captures the dataset characteristics for the anomaly prediction problem with high fidelity. Additionally, the SOTA results clearly demonstrate that “one-hot” encoding does not achieve a satisfactory performance.

### 4.4. Task Description

At each time step *t*, an anomaly $a^{t}\in A$ either occurs or does not. The goal is to predict measurements $M^{t}=\left\{m_{1}^{t},m_{2}^{t},\ldots ,m_{20}^{t}\right\}$ and the token assignment of the anomaly type $token(a^{t})$ at time step *t* (*two-dimensional* prediction). This prediction is performed multiple times, and the evaluation metrics are recorded.

## 5. The RI2AP Method

**Figure 2:**
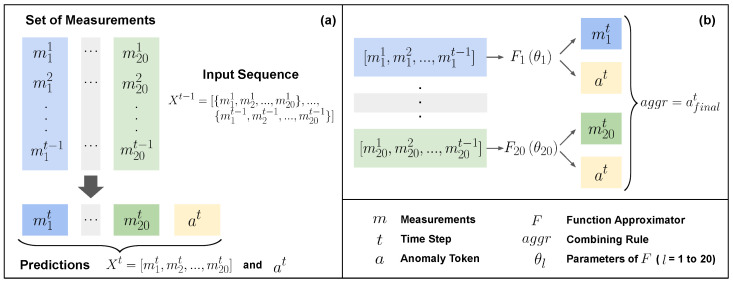
Illustrates the RI2AP method. (**a**,**b**) correspond to Equations (1) and (2), respectively.

**Figure 3:**
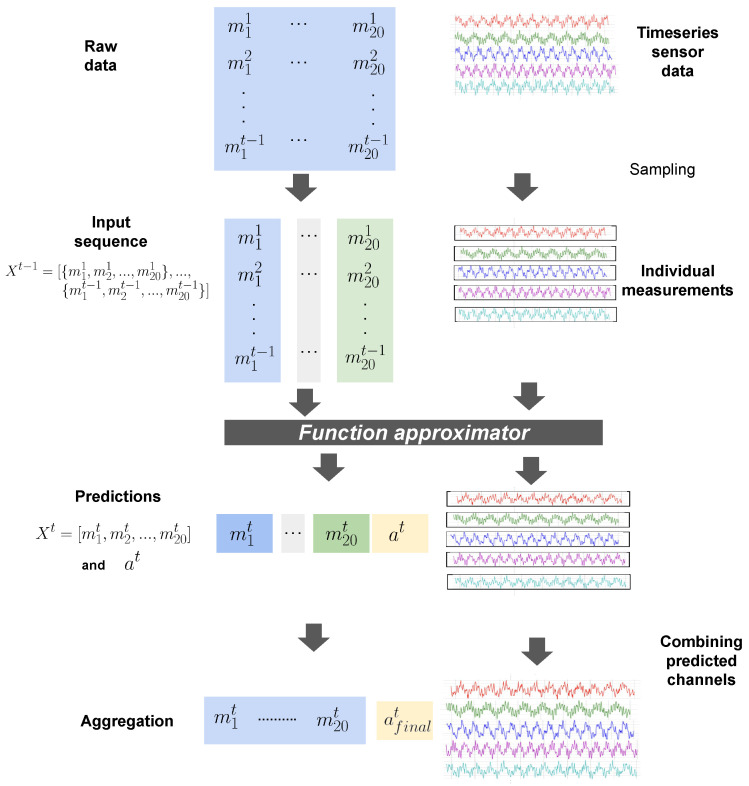
Detailed illustration of RI2AP.

In this section, we will first describe the RI2AP method (illustrated in Figure 2 and Figure 3), subsequently explain the motivations for the method design, and finally elaborate on the detailed model architecture used in the RI2AP method. Consider a series of measurements up to time step t−1, denoted by the data list $X^{t-1}=\left[M^{1}=\left\{m_{1}^{1},m_{2}^{1},\ldots ,m_{20}^{1}\right\},\ldots ,M^{t-1}=\left\{m_{1}^{t-1},m_{2}^{t-1},\ldots ,m_{20}^{t-1}\right\}\right]$. Here, $M^{t}$ represents the set of all 20 measurements at time *t*, and each of the $m_{l}^{t}$ represents one of these measurements at time step *t*. We first construct a set of 20 different function approximators from these measurements:

(1) $$\left\{F_{l}:\left(m_{l}^{1},\ldots ,m_{l}^{t-1};\theta_{l}\right)\to \left(m_{l}^{t},token\left(a^{t}\right)\right)|l\in \left[1,..,20\right]\right\}$$

$F_{l}$ represents a function approximator and is associated with a specific measurement $m_{l}^{t}$ at time step *t*. There are 20 such function approximators, indexed by *l* from 1 to 20. $\theta_{l}$ represents the parameters associated with the *l*th function approximation $F_{1}$. Here, the parameters are learned during the training process and are used to transform the input measurements into the predicted measurement $m_{l}^{t}$ and an associated token $token(a^{t})$. Then, we combine the set of all 20 outputs from each of the $F_{l}$ using a *combining rule* denoted as aggr to yield a final value $a_{final}^{t}$ [31]. This operation is described using the equation

(2) $$a_{final}^{t}=aggr\left(\left\{\left(m_{l}^{t},token\left(a^{t}\right)\right)|l\in \left[1,..,20\right]\right\}\right)$$

### 5.1. Design Motivations

#### 5.1.1. Why Separate Function Approximators and Combining Rules?

When domain experts analyze sensor measurements to understand their influence on the presence or absence of detected anomalies (typically conducted post anomaly occurrence), they initially examine the impacts of individual measurements separately. This approach stems from the fact that each measurement can strongly correlate independently with anomaly occurrences. An anomaly typically occurs when multiple measurements independently combine, with well-defined aggregation effects, to *cause* the anomaly. Due to this reason, we employ combining rules introduced in the independence of the causal influence framework [32], specifically designed for such use cases. These rules provide a natural and domain-expert-friendly way to express realistic aggregation effects, offering options like a simple OR, Noisy-OR, Noisy-MAX, tree-structured context-specific influences, etc, leading to enhanced interpetability. Additionally, as combining rules inherently represent compactly structured Bayesian networks, methods from the *do-calculus* can be applied to isolate and study various combinations of anomaly-causation models, making them uniquely suitable for our use case [33,34].

#### 5.1.2. Why Not Standard XAI Methods?

As briefly alluded to in Section 1, a qualitative issue with XAI methods is that they are primarily useful to ML researchers to gain insights into model behaviors and require some postprocessing or organization before end-users or domain experts can understand the model outcomes. They are *developer friendly* and not *domain expert friendly*. Additionally, there are also mathematical instability issues with XAI methods that raise questions about the robustness and reliability of the explanations provided. Specifically, XAI techniques are based on approximating the underlying manifold using a simpler surrogate, e.g., approximating a globally complex and non-linear function with a linear (LIME) or fixed-width kernel method (SHAP) for a particular test instance of interest [35,36]. This surrogate model needs training using a *representative* set, a challenging proposition to ensure in cases with class *rarity* such as anomaly prediction, resulting in surrogate model variability (producing different explanations for the same prediction when different instances of the surrogate model are applied) [37].

The combining-rules approach used in our work is readily interpretable by the domain expert due to its natural functional forms. Second, it comes with the calibration advantages of probabilistic models—predicted probabilities can be well calibrated to align with experimental observations due to factors that facilitate robustness, e.g., Bayesian estimation, do-calculus, uncertainty modeling, and model interpretability.

### 5.2. Function Approximation Methods

Section 5, Equation (1) introduced the general form for the function approximation used in the RI2AP method. For ease of the explanation of the architecture, we will consider the function approximation architecture corresponding to measurement *l*, given by

(3) $$F_{l}:\left(m_{l}^{1},\ldots ,m_{l}^{t-1};\theta_{l}\right)\to \left(m_{l}^{t},token\left(a^{t}\right)\right)$$

This model parameterized by $\theta_{l}$ takes as input the data list $X_{l}=\left[m_{l}^{1},\ldots ,m_{l}^{t-1}\right]$, i.e., the measurements corresponding to *l* up to time step t−1, and produces the output $\left(m_{l}^{t},token\left(a^{t}\right)\right)$, i.e., the measurement value and the anomaly type $token(a^{t})$ at time step *t*.

#### 5.2.1. Long Short-Term Memory Networks (LSTMs)

A natural choice for such a time step-dependent prediction scenario is any recurrent neural network (RNN)-based method modified to emit two-dimensional outputs [38]. The set of equations below describes an abstraction of the LSTM modified for our setting:

(4) $$\begin{array}{cc} \; & F_{l}:{\mathrm{LSTMCells}}_{1}^{t-1}\left(\left[m_{l}^{1},\ldots ,m_{l}^{t-1}\right];\theta_{l},H\right)\\ & \;\;\;\;\to {\mathrm{LSTM}}_{\mathrm{output}}=\left(m_{l}^{t},token\left(a^{t}\right)\right) \end{array}$$

Here, H denotes the hyperparameters such as choice of optimizer, learning rate scheduler, number of epochs, batch size, number of hidden layers, and dropout rate.

#### 5.2.2. Transformer Architecture—Decoder Only

The current SOTA model in RNN-based models is the Transformer architecture, which has been employed successfully in a wide variety of application domains [39]. We used two types of Transformer architectures in our experimentation: (i) our own decoder-only implementation modified to produce two-dimensional outputs at each autoregressive step [40] and (ii) TimeGPT [16], a foundational time series Transformer model.

The set of equations below describes an abstraction of the decoder-only Transformer architecture modified for our setting:

(5) $$\begin{array}{cc} \; & F_{l}:{{\mathrm{Transformer}}_{\mathrm{Blocks}}}_{1}^{B}\left(\left[m_{l}^{1},\ldots ,m_{l}^{t-1}\right];\theta_{l},{\mathrm{Attn}}_{\mathrm{Mask}},H\right)\\ & \;\;\;\;\to {\mathrm{Transformer}}_{\mathrm{output}}=\left(m_{l}^{t},token\left(a^{t}\right)\right) \end{array}$$

Here, ${\mathrm{Attn}}_{\mathrm{Mask}}$ denotes the attention mask required for the autoregressive decoder-only architecture (to prevent it from looking at future parts of the input when generating each part of the output). *B* represents the number of Transformer blocks. H denotes the hyperparameters such as choice of optimizer, learning rate scheduler, number of epochs, batch size, number of feedforward layers (with default hidden layer size), number of blocks, number of attention heads, and dropout rate.

#### 5.2.3. Method of Moments

In “A Kernel Two-Sample Test for Functional Data”, Wynee et al. [41] demonstrated that when comparing data samples with imbalanced sizes, using first-order *moments*, specifically sample means, is more suitable as a feature to identify discriminatory patterns. Intuitively, employing sample means or *averages* helps alleviate the impact of significant differences in sample sizes. Narayanan et al. [42] leveraged ideas from Shohat and Tamarkin’s book and generalized this idea to *n*th-order moments, providing theoretical proof and experimental observations that validate the method’s robustness to sample imbalances [43,44]. Let $\mathrm{moments}(\left[m_{l}^{1},\ldots ,m_{l}^{t-1}\right])$ denote the moments of the input list. The set of equations below describes an abstraction of the method of moments for our setting:

(6) $$\begin{array}{cc} \; & \mathrm{moments}(\left[m_{l}^{1},\ldots ,m_{l}^{t-1}\right];\mathrm{NN}\left(\theta_{NN},H\right))\\ \; & =E\left[[\mathrm{NN}{\left(\left[m_{l}^{1},\ldots ,m_{l}^{t-1}\right]\right)}^{1},\ldots ,\mathrm{NN}{\left(\left[m_{l}^{1},\ldots ,m_{l}^{t-1}\right]\right)}^{n}]\right],\\ \; & \mathrm{NN}{\left(\left[m_{l}^{1},\ldots ,m_{l}^{t-1}\right]\right)}^{j}\in R^{D\times 2},\;\forall j\in \left\{1,\ldots ,n\right\} \end{array}$$

where NN denotes a feedforward neural network that encodes the measurements at different time steps into a dense matrix of size D×2 (*D* is the output dimension of the penultimate layer of the neural network). $\theta_{NN}$ denotes the parameters of the network, H denotes the hyperparameters, such as the number of hidden layers and their sizes, and *n* denotes the $n^{\mathrm{th}}$ order moment. The reason for the neural network in this setup is to be able to learn a mapping from the inputs to a transformed basis, over which the moments are calculated. For a normally distributed sample, it is clear that the first- and second-order moments (mean and variance) of the measurements (before transformation to any other basis) are *sufficient* to characterize the distribution. However, in our case, the underlying data distribution is unknown. Therefore, we equip the function approximator with a neural network that can be trained to map inputs to a transformed basis, ensuring that the calculated moments sufficiently characterize the distribution.

*We chose the function approximator choices of the LSTM and Transformer models as they represent the SOTA models in sequence modeling. We chose the method of moments due to its ideal theoretical properties (robustness to noise and class imbalance) with respect to our problem setting.*

## 6. Experiments and Results

### 6.1. Function Approximator Setup Details

#### 6.1.1. LSTM

The preprocessed dataset was divided into training and testing sets, with the training set encompassing the initial 80% of the temporal data, and the remaining 20% allocated to the test set. Sequences were constructed from the normalized data utilizing a look-back length (context window) of 120. We used PyTorch Lightning’s Trainer to train and validate the model. The training process was set up with the Mean Squared Error (MSE) loss function, and the AdamW optimizer, with its learning rate scheduler. The hyperparameters tuned included the number of epochs, batch size, hidden layers, and dropout rate. Early stopping was implemented, and the best checkpoint, determined by the reduction in MSE, was saved during training to monitor the validation loss.

#### 6.1.2. Transformer (Ours)

As mentioned in Section 5, we implemented our own decoder-only Transformer setup. The preprocessed data were split into training and testing sets using the same splitting method used for the LSTM in the section. Subsequently, the data were normalized and transformed into sequences of a look-back length of 120 for training the Transformer model. Once again, the training process was set up with an MSE loss function, AdamW optimizer, and a learning rate scheduler. The model was trained and validated using PyTorch Lightning’s Trainer module, with early stopping implemented to prevent overfitting. The training progress was monitored and logged, and the best model checkpoint was saved based on the validation loss. The model’s hyperparameters included the number of epochs, batch size, number of feedforward layers (with a default hidden layer size = 2048), number of blocks = 6, number of attention heads, and dropout rate.

#### 6.1.3. TimeGPT

The dataset was preprocessed before being divided into two subsets, training and testing, each with 97,510 and 2000 rows. Both sets were then standardized using a standard scaler. The training of the model was performed using the *timegpt.forecast* method, and hyperparameter tuning was performed using *finetune_steps*, which performs a certain number of training iterations on our input data and minimizes the forecasting error. However, given Nixtla’s current constraints, more hyperparameter tuning beyond *finetune_steps*, such as modifying the learning rate, batch size, or dropout layers, was not possible due to a lack of precise insights into the model’s architecture. It is worth noting that the TimeGPT SDK and API have no restrictions on dataset size if a distributed backend is used. Other essential parameters used in the model included the *frequency*, *level*, *horizon*, *target column*, and *time* column. More information is provided in Appendix D.

#### 6.1.4. Method of Moments

The preprocessing steps are similar to the LSTM and Transformer cases. The order of moments *n* was taken as 2 (starting from 0), and the number of hidden layers in the neural network NN were 2. The loss function was MSE, and the optimizer used was AdamW. Root Mean Squared Error (RMSE) scores were calculated for the predictions, and the best-performing checkpoint was stored (best performing in terms of training loss). The training progress was monitored and logged, and the best model checkpoint was saved based on the validation loss.

We will now report the evaluation results. Table 3 provides a list of abbreviations, which we use in the result tables.

### 6.2. Evaluation Results Using Individual Measurements

We present Mean Squared Error (MSE) values and additionally categorize regression values based on token assignment, aligning them with the closest ground truth values. This categorization is crucial for computing traditional classification-based metrics, enhancing the interpretability of results for domain experts. The precision, recall, F1 score, and accuracy results for the LSTM and Transformer are detailed in Table 4. RMSE and MSE comparison results are provided in Table 5 in the same section. Table 6 summarizes aggregated measurements for all anomaly types. Notably, the TimeGPT model performed poorly; however, it is important to highlight that we lacked access to the model for fine-tuning on our dataset. The LSTM model outperformed the Transformer, possibly due to Transformers losing temporal information and facing overfitting issues related to the quadratic complexity of attention computation [45,46,47]. The method of moments demonstrates a significantly better performance among function approximators, supporting our expectation that it is particularly well suited for robust anomaly prediction within the experimental context of this study.

### 6.3. Evaluation Results with Combining Rules

We used two separate combining rules, Noisy-OR and Noisy-MAX, as introduced in the independence of the causal influence framework [20]. Combining rules combines probability values and not regression values. Therefore, we used the sigmoid of the binned regression values (binned to the closest token assignment) to convert the closeness value into a number between 0 and 1. This number denotes the probability of the influence of the corresponding measurement on the prediction outcome.

**Figure 4:**
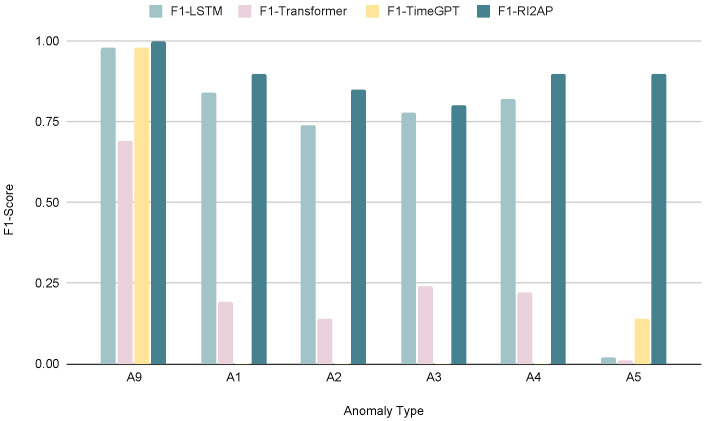
Comparison of F1 score with the LSTM model, Transformer, and the method of moments using Noisy-OR. (A1:A5, A9—see Table 3).

**Figure 5:**
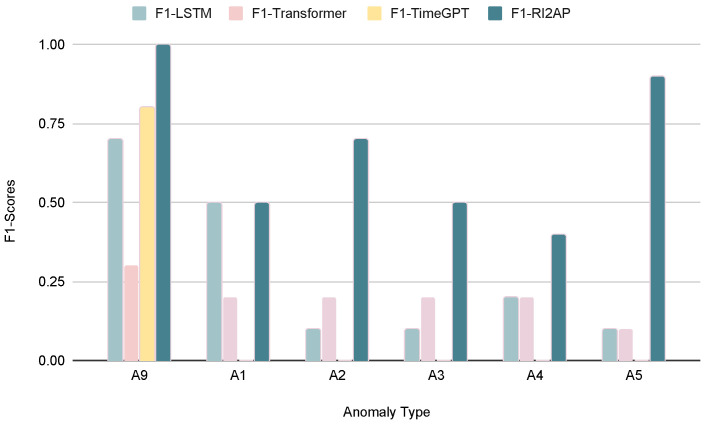
Comparison of F1 score with the LSTM, Transformer, and the method of moments using Noisy-MAX. (A1:A5, A9—see Table 3).

**Figure 6:**
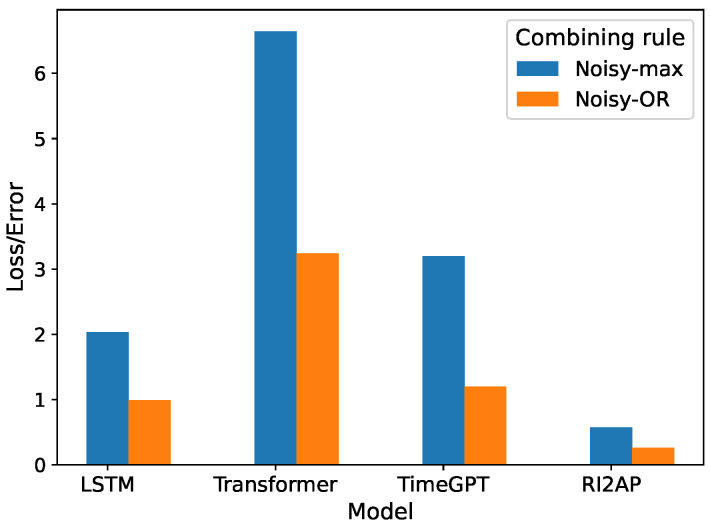
Loss/error comparison of different function approximator choices and combining rule predictions.

The precision, recall, and F1 measures of the LSTM and Transformer models with the Noisy-OR combining rule are reported in Table 7, and those for the Noisy-MAX combining rule in Table 8. Here, we notice that the Noisy-OR rule results in better predictions compared to the Noisy-MAX rule. This shows that the severity of the anomalous occurrence compounds with multiple failing parts and does not depend on any single critical part failure (recall the illustration from Figure 1). The precision, recall, and F1 measures for the method of moments with Noisy-OR and Noisy-MAX are shown in Table 9. As expected again, the method of moments achieved superior results in predicting anomalies. We also examined how different anomaly types with varying rarities affect the performances of different models. The findings demonstrate that regardless of the rarity of an anomaly, the method of moments outperformed the other function approximator choices, which, in contrast, exhibit results with significant variance, as shown in Figure 4 and Figure 5. A comparison of RMSEs among the different function approximator choices using the combining rules is presented in Table 10 and Figure 6. Consistently, the method of moments exhibited a superior performance, showing lower RMSE values compared to other function approximators. This reaffirms its predictive effectiveness, particularly in addressing the infrequent occurrence of anomalies.

**Table 4:** Evaluation results of baselines in univariate predictions: precision, recall, F1 score, and accuracy ^∗^.

B	LSTM				Transformer				TimeGPT			
**S**	42,189				42,299				2000			
**M**	P	R	F1	A	P	R	F1	A	P	R	F1	A
V1	0.2	0.3	0.3	0.7	0.2	0.3	0.2	0.2	0.5	0.5	0.5	0.9
V2	0.8	0.7	0.8	0.9	0.2	0.1	0.2	0.3	0.5	0.5	0.5	0.9
V3	0.9	0.8	0.9	0.9	0.2	0.3	0.2	0.5	0.5	0.5	0.5	1
V4	0.9	0.9	0.9	0.9	0.2	0.1	0.1	0.1	0.5	0.5	0.5	1
V5	1	1	1	1	0	0.1	0	0.1	0.2	0.1	0.1	0.8
V6	0.9	0.9	0.9	0.9	0.2	0.2	0.1	0.5	0.5	0.5	0.5	0.9
V7	0.9	0.9	0.9	0.9	0.2	0.3	0.2	0.6	0.2	0.1	0.16	0.9
V8	0.9	1	1	1	0.4	0.4	0.4	0.7	0.5	0.5	0.5	1
V9	1	0.9	0.9	1	0.2	0.3	0.3	0.6	0.3	0.3	0.3	0.9
V10	0.9	0.9	0.9	0.9	0.3	0.3	0.2	0.5	0.2	0.1	0.1	0.7
V11	0.9	0.9	0.9	0.9	0	0.1	0	0	0.2	0.2	0.8	0.9
V12	0.4	0.5	0.3	0.5	0.4	0.3	0.4	0.7	1	1	1	1
V13	0.7	0.7	0.7	0.9	0.3	0.4	0.4	0.6	0.5	0.5	0.5	1
V14	0.9	0.9	0.9	1	0.6	0.7	0.5	0.7	0.5	0.5	0.5	0.9
V15	1	1	1	1	0.2	0.3	0.1	0.5	0.3	0.3	0.3	1
V16	0.8	0.7	0.7	0.8	0.2	0.2	0.2	0.4	0.2	0.2	0.2	0.9
V17	0.9	0.9	0.9	0.9	0.3	0.4	0.3	0.6	0.5	0.5	0.5	0.9
V18	1	1	1	1	0.2	0.2	0	0.1	0.5	0.5	0.5	1
V19	0.9	0.9	0.9	0.9	0.3	0.4	0.3	0.2	0.5	0.5	0.5	0.9
V20	0.9	0.9	0.9	0.9	0.9	0.9	0.9	0.9	0.5	0.5	0.5	0.9

^∗^. B—Baseline models, S—Support, M—Performance metric, P—Precision, R—Recall, F1—F1 Score, A—Accuracy, V1:V20—see Table 3.

**Table 5:** Evaluation results of baselines in univariate predictions: RMSE and MSE ^∗^.

B	LSTM			Transformer			TimeGPT		
**S**	42,189			42,189			2000		
**M**	$R^{\prime }$	$M^{\prime }$	${\mathrm{TR}}^{\prime }$	$R^{\prime }$	$M^{\prime }$	${\mathrm{TR}}^{\prime }$	$R^{\prime }$	$M^{\prime }$	${\mathrm{TR}}^{\prime }$
V1	0.7	0.5	0.7	6.4	41.1	29.2	1.1	1.2	1.6
D	0.6	0.4		1.3	1.8		1.9	3.7	
V2	0.7	0.5	0.7	0.5	0.3	2015.4	1.7	2.9	1.3
D	0.8	0.6		0.6	0.4		0.8	0.7	
V3	0.8	0.6	0.7	3.6	13.2	9.8	1.1	1.2	0.8
D	0.4	0.2		2.09	4.2		0	0	
V4	1	1	0.7	4.1	17.1	12.4	1.2	1.5	0.9
D	0.2	0.4		1.9	3.8		0.3	0.1	
V5	0.4	0.2	0.5	2.7	6.9	5.7	1	1	1.7
D	0.6	0.4		1.8	3.5		2.2	4.7	
V6	0.6	0.4	0.6	5.8	33.9	24.1	1	1	0.8
D	0.5	0.3		1.8	3.4		0.5	0.3	
V7	0.6	0.4	0.6	4.6	22	16	1	1	1.3
D	0.5	0.2		1.4	2		1.6	2.6	
V8	0.1	0.1	0.1	0	0	2.8	1	1.1	0.7
D	0.1	0.1		1	1		0.2	0	
V9	0.9	0.9	0.8	6.4	41.2	29	1	1	1.4
D	0.4	0.1		1.4	2		1.7	2.8	
V10	0.5	0.2	0.5	5.6	32.3	23	1	1	2.3
D	0.5	0.2		1.6	2.6		3.1	9.9	
V11	0.9	0.8	0.7	2.6	6.9	16.5	1.3	1.7	1.3
D	0.2	0.1		1.8	3.5		0.9	0.9	
V12	0.9	0.8	1.3	9.2	85.1	60.3	1.3	1.6	1.2
D	1.7	2.9		1.04	1.1		0	0	
V13	1	1	0.7	7.1	50.5	35.8	1.1	1.3	0.9
D	0.4	0.2		1	1		0.5	0.2	
V14	0.9	0.7	0.7	7.6	58	41	4.1	16.6	3.1
D	0.3	0.1		1.1	1.4		0.7	0.5	
V15	1	0.9	0.7	57.2	3274	2315.1	1	1	0.8
D	0.2	0		1.7	3		0.3	0.1	
V16	1.1	1.1	1	6.4	41	29	2.1	4.2	1.6
D	0.8	0.7		1.7	3		0.8	0.7	
V17	0.8	0.6	0.6	56.2	3166.8	2239.2	0.8	0.7	0.9
D	0.4	0.2		1.4	2		0.8	0.6	
V18	0.4	0.1	0.5	30.7	948	670.3	1	1	0.7
D	0.5	0.3		2.05	4.2		0.2	0	
V19	0.6	0.4	0.7	46.3	2141.8	1514.4	0.9	0.8	0.9
D	0.8	0.7		5.9	3.5		0.7	0.4	
V20	0.8	0.6	0.6	36.8	1352.1	956.1	1	1.1	0.9
D	0.3	0		2.6	6.7		0.8	0.6	

^∗^. B—Baseline models, S—Support, M—Performance metric, $R^{\prime }$—RMSE, $M^{\prime }$—MSE, ${\mathrm{TR}}^{\prime }$—Total RMSE, V1:V20—see Table 3.

**Table 6:** Evaluation results of RI2AP: precision, recall, F1 score, and accuracy ^∗^.

Model	RI2AP			
**S**	11,927			
**M**	P	R	F1	A
V1	0.6	1	0.7	0.7
V2	1	1	1	1
V3	1	1	1	1
V4	1	1	1	1
V5	0.8	1	0.9	0.9
V6	0.7	0.8	1	0.8
V7	1	1	1	1
V8	0.8	1	0.9	0.8
V9	1	1	1	1
V10	0.8	1	0.9	0.8
V11	1	1	1	1
V12	1	1	1	1
V13	0.8	1	0.9	0.8
V14	1	1	1	1
V15	0.8	1	0.9	0.8
V16	0.8	0.9	1	0.8
V17	1	1	1	1
V18	1	1	1	1
V19	1	1	1	1
V20	1	1	1	1

^∗^. S—Support, M—Performance metric, P—Precision, R—Recall, F1—F1 Score, A—Accuracy, V1:V20—see Table 3.

**Table 7:** Evaluation results: Noisy-OR results of LSTM and Transformer ^∗^.

AT	LSTM				Transformer				TimeGPT			
	P	R	F1	A	P	R	F1	A	P	R	F1	A
A9	0.9	0.9	0.9	0.9	0.6	0.8	0.7	0.4	0.9	1	0.9	0.9
	S-382328						S-38465					
A1	0.9	0.8	0.8		0.2	0.3	0.2		0	0	0	0
	S-224081						S-699					
A2	0.7	0.8	0.7		0.2	0.1	0.2		0	0	0	0
	S-77556						S-237					
A3	0.7	0.9	0.8		0.3	0.2	0.2		0	0	0	0
	S-89768						S-717					
A4	0.8	0.8	0.8		0.3	0.2	0.2		0	0	0	0
	S-64471						S-169					
A5	0.1	0.1	0.1		0.3	0.1	0.1		0	0	0	0
	S-5576						S-313					
**MA**	0.7	0.7	0.7		0.3	0.3	0.3		0.2	0.2	0.2	

^∗^. AT—Anomaly type, P—Precision, R—Recall, F1—F1 Score, A—Accuracy, S—Support, MA—Macro average, A1:A9—see Table 3.

**Table 8:** Evaluation results: Noisy-MAX results of baselines ^∗^.

AT	LSTM				Transformer				TimeGPT			
	P	R	F1	A	P	R	F1	A	P	R	F1	A
A9	0.7	1	0.8	0.5	0.3	0.4	0.3	0.4	0.7	1	0.8	0.7
	S-12626						S-1354					
A1	0.5	0.4	0.5		0.2	0.3	0.2		0	0	0	0
	S-11377						S-348					
A2	0.1	0.1	0.1		0.2	0.1	0.2		0	0	0	0
	S-3664						S-113					
A3	0.1	0.1	0.1		0.2	0.1	0.2		0	0	0	0
	S-4170						S-56					
A4	0.2	0.3	0.3		0.2	0.2	0.2		0	0	0	0
	S-6762						S-22					
A5	0.1	0.1	0.1		0.2	0.1	0.1		0	0	0	0
	S-3590						S-107					
**MA**	0.4	0.5	0.4		0.2	0.2	0.2		0.2	0.2	0.2	

^∗^. AT—Anomaly type, P—Precision, R—Recall, F1—F1 Score, A—Accuracy, S—Support, MA—Macro average, A1:A9—see Table 3.

**Table 9:** Evaluation results: Noisy-OR and Noisy-MAX results of RI2AP ^∗^.

AT	Noisy-OR					Noisy-MAX				
	P	R	F1	A	S	P	R	F1	A	S
A9	1	1	1	0.8	2000	1	1	1	0.6	100
A6	0.5	0.6	0.5		1600	0.4	0.5	0.5		100
A1	0.8	0.8	0.9		1989	0.4	0.5	0.5		100
A2	0.8	0.8	0.8		2011	1	0.5	0.7		200
A3	0.9	0.6	0.8		2800	0.3	0.4	0.5		53
A7	1	0.9	1		2200	0.6	0.4	0.5		82
A4	0.9	0.9	0.9		1800	0.5	0.4	0.4		65
A5	0.9	0.9	0.9		1900	1	0.9	0.9		100
A8	1	0.9	1		2100	1	0.9	1		100
TS					18,000					900

^∗^. AT—Anomaly type, P—Precision, R—Recall, F1—F1 Score, A—Accuracy, S—Support, TS: Total Support, A1:A9—see Table 3.

**Table 10:** Evaluation results: combining rules (RMSE).

Baseline	Noisy-Max	Noisy-OR
LSTM	2.04	0.99
Transformer	6.64	3.24
TimeGPT	3.2	1.19
**RI2AP**	**0.57**	**0.23**

## 7. Deployment of RI2AP

**Figure A4:**
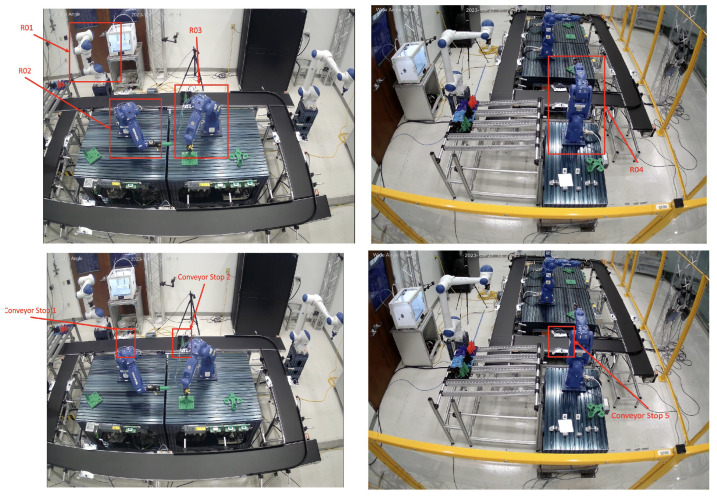
Some images from FF Cell: R01—Robot 1, R02—Robot 2, R03—Robot 3, R04—Robot 4.

The deployment of the proposed RI2AP method was carried out in the Future Factories cell, which is shown in Figure A4. The deployment plan, technical details, results, and issues faced in deployment are as follows.

### 7.1. Deployment Plan

1. Input: The first step involves gathering and organizing saved models for important sensor variables, ensuring that they are ready for deployment. These saved models constitute the baselines and the proposed linear model based on the method of moments. An important task in this step is to verify the availability and compatibility of these models to be deployed in the FF setup.
2. Data Preparation: This step involves integrating real-time data with server and Program Logic Controller (PLC) devices, enabling the collection of real-time data for analysis. Anomaly simulation mechanisms were developed to simulate various anomalies in the FF cell, tailored to each modeling approach, while normal event simulation was also conducted for training and testing purposes.
3. Experimentation: This step involves feeding the prepared real-time data into the baseline models to analyze and predict outcomes.
4. Output: The output includes generating predictions for normal and anomalous events in the future based on the deployed models.
5. Validation: The validation of the results was carried out through expert validation, where domain experts in the FF lab validated the results obtained from the deployed models. The predictions were cross-checked with findings from previous research or empirical observations to ensure their accuracy and reliability.
6. Refinement: The refinement of the models was undertaken based on validation results and feedback from domain experts, ensuring that the deployed models were effective and accurate. An iterative improvement process was implemented, involving refinement, testing, and validation cycles to continually enhance the effectiveness and accuracy of the deployed models.

### 7.2. Technical Details of Deployment

With an abundance of industrial communication protocols available within manufacturing systems, a successful deployment strategy hinges upon utilizing the correct technologies to enable the proper functioning of the trained model. The Future Factories cell has two main communication protocols utilized throughout the equipment. The first uses MQTT as the main pathway to send and receive data. This is performed by collecting the data on an edge device present within the cell and publishing the data to a public MQTT broker for different assets to access. However, since this method utilizes a public broker, the lag increases between the time it is generated and the time it is received.

**Figure 7:**
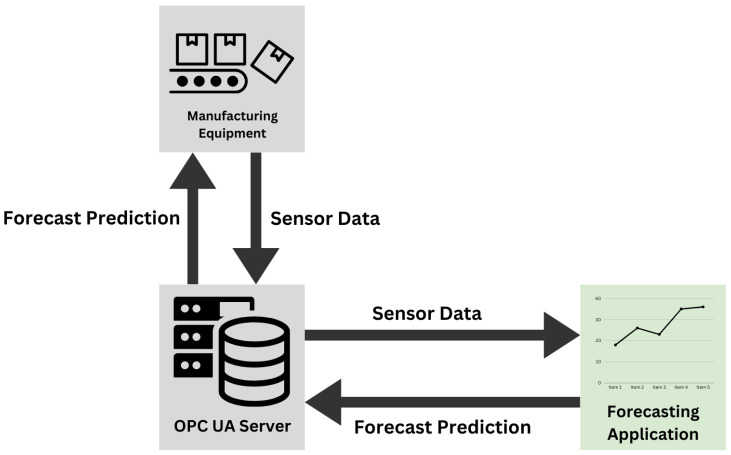
Deployment architecture of forecasting model.

To ensure that the model operates as intended, it must receive data as near to real time as possible. As such, the MQTT pathway might introduce some errors in the forecasting timing. The other data pathway available utilizes OPC UA. In this option, the PLC present in the system hosts a local OPC UA server that receives data from the PLC every 10 ms and broadcasts them to any client connected to the server. As such, this path presents a more adequate solution. The full deployment architecture can be seen in Figure 7. In this architecture, the trained model is deployed on a separate machine connected to the same network as the OPC UA server. The machine hosts an application that searches for the required data tags in the OPC UA information model and feeds them into the model. Once the next time step is predicted, it can be relayed back to the system through the server as well and take any corrective actions if needed.

### 7.3. Results of Deployment and Discussion

**Figure 8:**
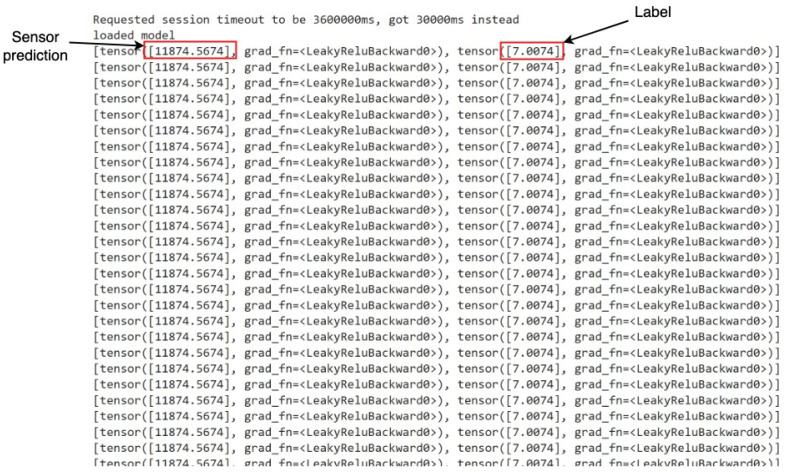
Deployment Result 1—Potentiometer R02 Sensor and Anomaly type: Body2Removed.

**Figure 9:**
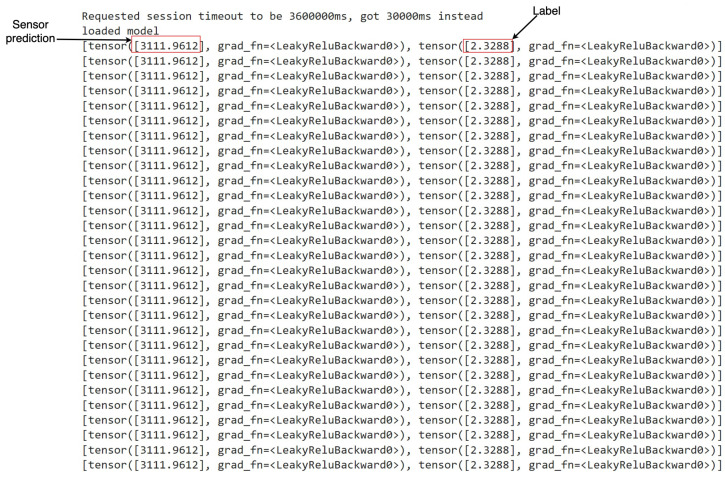
Deployment Result 2—Potentiometer R03 Sensor and Anomaly type: R04 crashed nose.

During the deployment phase, various types of anomalies, as outlined in Table 2, were systematically simulated to evaluate the efficacy of the deployed models. Data generated from relevant robots and sensors, capable of capturing these anomalies within the assembly pipeline, were fed into the models developed through the R12AP training process. Representative illustrations depicting this process are presented in Figure 8 and Figure 9. This methodology facilitated the comprehensive testing and validation of the deployed system’s capability to predict and respond to diverse anomaly scenarios within the manufacturing environment, thereby ensuring its robustness and reliability in practical applications.

In Figure 8 and Figure 9, denoted as *Sensor prediction* and *Label*, respectively, we delineate the essence of our predictive models’ output. The former signifies the projected next sensor reading, while the latter distinguishes between anomalous and normal states. Upon reviewing the snapshots, an observation arises: there are instances where the model flags a state as anomalous despite the absence of any actual anomaly.

The reason for false predictions is analyzable vis-a-vis the parameters of the combining rule. Specifically, the lack of intricate contextual interactions between the predictor variables (the multiple sensors) is omitted due to their separation during modeling, resulting in an insufficient understanding of the status of the system. However, the causal-influence framework allows natural extensions to other dependency structures (relating the influence among the multiple sensors), such as general Directed Acyclic Graph (DAG) forms. It is evident that our proposed methodology *RI2AP*, employing combining rules, represents a more sufficient solution in this context, primarily due to its enhanced interpretability and alignment with domain experts’ requirements.

We plan to pursue this avenue in proposing a solution to this issue. The advantage of our framework is that it helps inform remedial measures during iterative development with the end goal of obtaining a robust deployment. The initial findings in deployment represent the nascent phase in a more extensive deployment regimen. Looking ahead, our further work in deployment entails implementing the combined model that we proposed, which will be tailored explicitly to the requirements of manufacturing environments.

### 7.4. Engineering Challenges Faced in Deployment

During the deployment of RI2AP within the real manufacturing environment at the FF laboratory, several challenges arose that required careful attention and resolution. Primarily, significant effort was dedicated to adapting the code to seamlessly align with the format of the input data stream, ensuring smooth integration and functionality. Additionally, sensor-related issues emerged, with some sensors failing to generate acceptable values, necessitating intervention from domain experts to troubleshoot and rectify the discrepancies. Another hurdle involved the simulation of anomalies, which posed difficulties in accurately replicating real-world scenarios. Moreover, the process of selecting suitable robots and sensor values for testing alongside simulated anomalies proved to be intricate, requiring close collaboration and expertise from the FF lab’s domain specialists to navigate effectively. Through concerted efforts and the expertise of the involved stakeholders, these challenges were addressed and managed, contributing to the advancement and refinement of RI2AP’s deployment in the manufacturing environment.

## 8. Conclusion, Future Work, and Broader Impact

This paper introduced a novel methodology, RI2AP, for anomaly prediction, designed to address unique challenges related to anomaly prediction in rocket assembly pipelines. We employed *combining rules* for an enhanced domain-expert-friendly interpretation of the results. Empirical evaluations demonstrated the effectiveness of our proposed methodology.

### 8.1. Future Work

Equipped with a proof-of-concept implementation of our proposed method, we will explore several enhancements in future work. Firstly, we will learn a *multisensor* function approximator that considers all 20 measurements simultaneously, utilizing a neural network, and track the performance gap between our current implementation and the multisensor model’s accuracy. This approach aims for precise quantification, balancing the trade-off between accuracy and interpretability while integrating multiple individual-function approximators. Secondly, we intend to investigate the impact of alternative combining rules, such as tree-structured conditional probability effects, and leverage do-calculus to manage potential backdoors and confounding factors. This step expands the exploration of combining rules beyond our current approach. Lastly, to enhance the interpretability of our methodology for domain experts, we propose developing higher-level representations of causal phenomena related to anomalies. This involves exploring connections between sensor measurements and high-level constructs (such as structural integrity or gripper failures), offering insights beyond ground-level sensor readings in understanding anomalous occurrences.

### 8.2. Broader Impact

While the focus of this paper has been the application of the RI2AP method to rocket assembly, the techniques proposed in this paper are fundamental and broadly applicable to other domains with similar problem characteristics, namely, rare-event categories, dependencies between events, and causal structures between factors affecting the rare events. Importantly, the proposed model was designed to be robust to inherent stochasticity (noise and anomalies) in processes that produce time series data collected from physical sensors and contain expressive mechanisms for deriving explanations (that support causality), facilitating insights that are readily interpretable by the end-user. Example applications include rare-event prediction in other manufacturing pipelines, corner-case prediction in healthcare applications (cases that deviate from the standard treatment protocol), etc. Finally, due to the unified handling of the causal-influence frameworks that adeptly deals with symbolic variables and powerful function approximation architectures that handle real-valued variables, natural extensions toward incorporating neuro-symbolic or generally statistical/symbolic/probabilistic approaches (with uncertainty estimation) are potentially promising avenues to explore.

## Figures and Tables

**Table 1:** FF Dataset and its statistics.

Dataset Artifact	Statistic
Rarity percentage	13.36%
Frequency	10 Hz
Data collection period	6 h
Original features	41
Selected features	20
Number of data points	211,546
Train/test split	80:20
Train samples	169,236
Test samples	42,309

**Table 2:** Anomaly types in FF Dataset.

Anomaly Type and Notation	Sub Type	Count	Percentage
Nosecone Removed	Type 1	9043	4.27%
BothBodies and Nose Removed	Type 3	4405	2.08%
TopBody and Nose Removed	Type 2	5904	2.79%
Body2 Removed	Type 1	3306	1.56%
Door2_TimedOut	Type 4	3711	1.75%
R04 crashed nose	Type 4	1631	0.77%
R03 crashed tail	Type 4	1426	0.67%
ESTOPPED	Type 4	273	0.13%
No anomaly	None	183,272	86.63%

**Table 3:** List of abbreviations.

Variable	Abbreviation	Variable	Abbreviation
Anomaly Label	D	LoadCell_R04	V15
SJointAngle_R03	V1	BJointAngle_R04	V16
Potentiometer_R04	V2	Potentiometer_R03	V17
VFD2	V3	Potentiometer_R01	V18
LoadCell_R02	V4	Potentiometer_R02	V19
LJointAngle_R01	V5	LoadCell_R03	V20
BJointAngle_R03	V6	Nosecone Removed	A1
UJointAngle_R03	V7	BothBodies and Removed	A2
VFD1	V8	TopBody and Nose Removed	A3
RJointAngle_R04	V9	Body2 Removed	A4
SJointAngle_R02	V10	Door2_TimedOut	A5
LJointAngle_R04	V11	R04 crashed nose	A6
SJointAngle_R04	V12	R03 crashed tail	A7
LoadCell_R01	V13	ESTOPPED	A8
TJointAngle_R04	V14	No anomaly	A9

**Figure A1:**
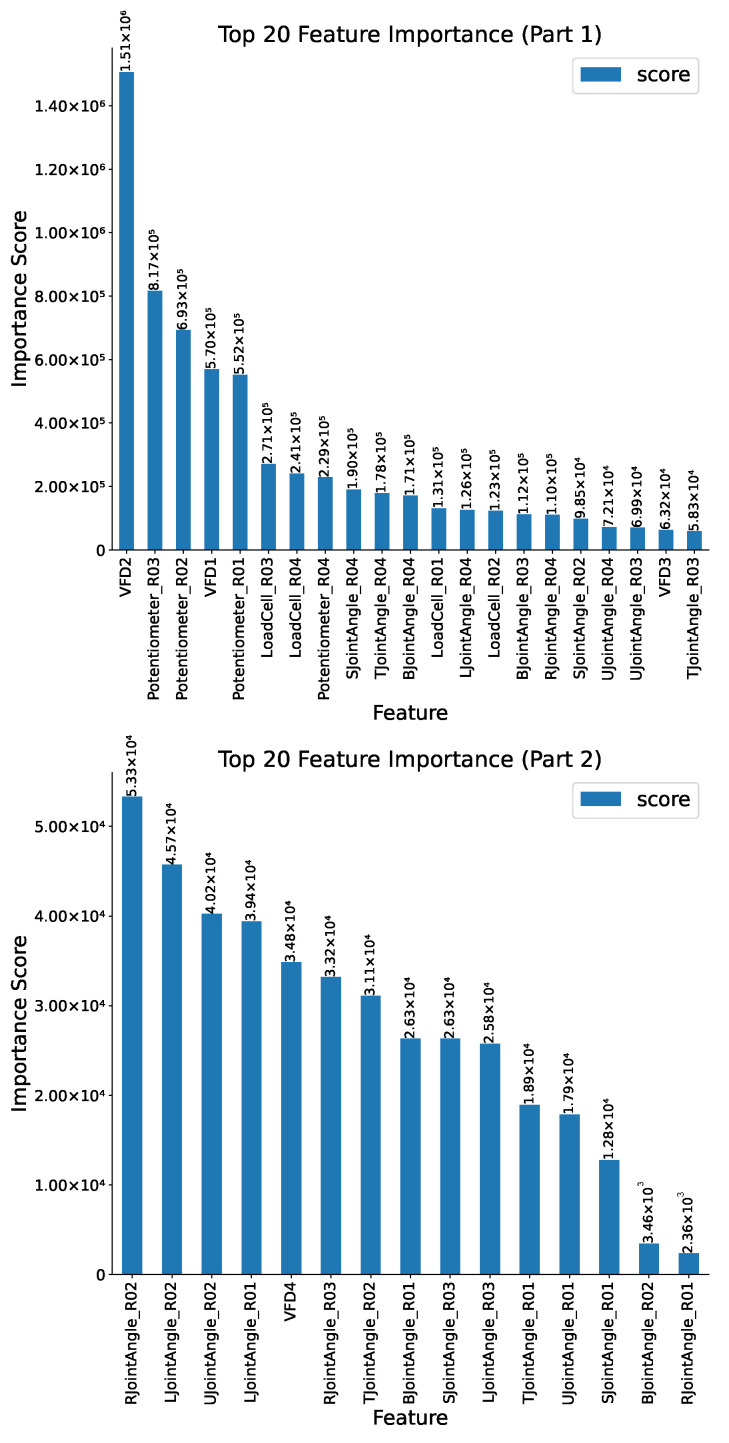
Feature importance scores using XGBoost *Cover* measure for all the features.

**Figure A2:**
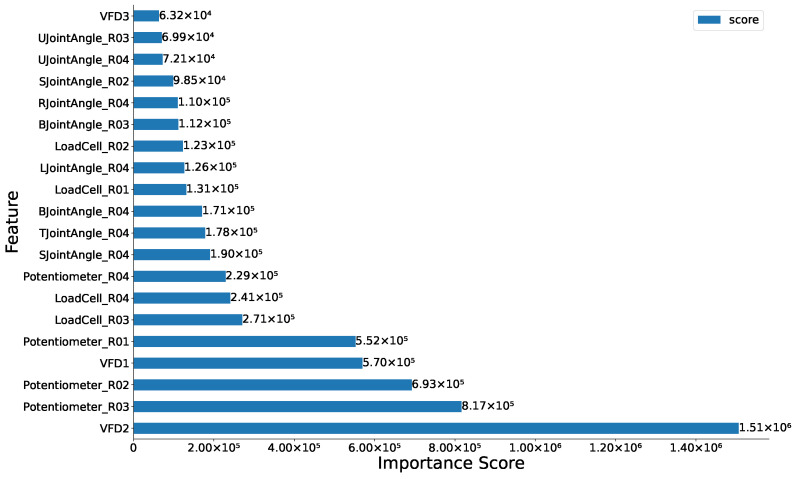
Feature importance scores of XGBoost *Cover* measure for top 20 features.

**Figure A3:**
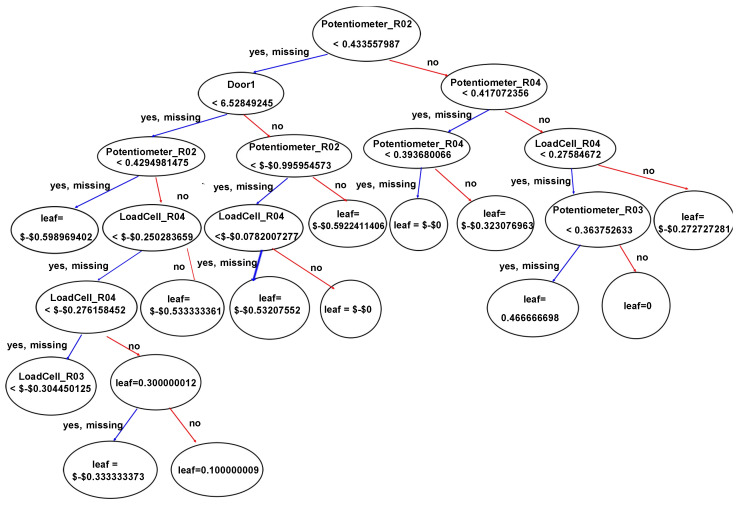
XGBoost tree.

## Data Availability

Code to reproduce the results is available at this link (https://github.com/ChathurangiShyalika/RI2AP, accessed on 15 May 2024).

## Appendix A. Supervised Learning Methods in Time Series Forecasting and Anomaly Detection and Prediction

Over the decades, the field of time series has witnessed the origin of various general forecasting methods for time series. These methods include traditional statistical approaches like Autoregressive Integrated Moving Average (ARIMA) [7], exponential smoothing [8], structural models [9], and Machine Learning (ML) approaches like Boosting algorithms [10], recurrent neural networks (RNN) [11], Long Short-Term Memory (LSTM) [12], and Deep Learning (DL)-based methods like convolutional neural networks (CNNs) [13]. More recently, there has been the invention of a surge array of Transformer-based models for time series forecasting [48,49,50,51]. Studies like [14,15] are some examples where Transformer-based models have been used in anomaly prediction.

While existing forecasting models, predicated on statistical, ML, and DL techniques, have demonstrated efficacy in certain contexts, the introduction of Transformer-based forecasting methods represents a contemporary shift in the landscape. However, it can be observed empirically that certain prior models like statistical approaches have exhibited superior performances compared with Transformers due to various limitations within the Transformers. In time series forecasting, Transformers suffer from issues like loss of time series temporal information, the quadratic complexity of sequence length, slow training and inference speed due to the encoder-decoder architecture, and overfitting issues [6,45,46,47]. Consequently, this investigation has further led researchers to explore linear forecasting-based methods [45] in forecasting, and they achieved more success compared with Transformer-based models.

Presently, Generative Artificial Intelligence has exhibited remarkable success, experiencing rapid advancements, particularly in the domains of Natural Language Processing (NLP) and Computer Vision (CV). Recent successes in Large Language Models (LLMs) in the above-mentioned domains have the potential to inspire a more comprehensive analysis of time series compared to traditional statistical methods, including ML and DL methods. The emergence of pre-trained foundational models and their immense success in NLP and CV has influenced the birth of pre-trained foundational models like TimeGPT [16], Lag-Llama [52], PreDcT [53], and PromptCast [17] for time series. Of these pre-trained models, TimeGPT [16] and PromptCast [17] have been focused on their applicability to anomaly detection and prediction.

## Appendix B. XGBoost Feature Coverage Plots

In our study, we initially employed XGBoost to assess the *total coverage* of features. Total coverage refers to the cumulative contribution of individual features toward the predictive performance of the model. XGBoost is the current SOTA algorithm for anomaly prediction. Given its efficacy, we leveraged XGBoost to obtain the importance of each feature and subsequently focused on the top 20 features based on their contributions. The decision to select a subset of features was rooted in the pragmatic necessity of managing dimensionality and enhancing model interpretability. For clarity and due to space constraints, we delineated the feature importance graph into two graphs, as shown in Figure A1. Furthermore, we explicitly highlight the top 20 features selected for further analysis, as shown in Figure A2. We also show the XGBoost tree in Figure A3, which shows the feature importance in the decreasing depth.

## Appendix C. Initial Experiments on Anomaly Detection and Anomaly Prediction

### Appendix C.1. Anomaly Detection

We conducted preliminary anomaly detection experiments using XGBoost to classify anomaly types, as detailed in Table A1. The original dataset was randomly split into training and testing sets with an 80:20 ratio. All 41 features from the original dataset were utilized, employing one-hot encoding, represented as the following dictionary. The results revealed a classification accuracy of 91%.

### Appendix C.2. Anomaly Prediction

Preliminary experiments for anomaly prediction were conducted utilizing XGBoost to classify events as anomalous or not (normal), as elaborated in Table A2. The initial dataset was randomly partitioned into training and testing sets at an 80:20 ratio. All 41 features from the original dataset were employed, and one-hot encoding was applied, representing 1 for anomalies and 0 for normal events. The prediction was made one minute in advance. The outcomes demonstrated a classification accuracy of 97%.

# *One* *hot* *encoded* *anomaly* *dictionary* labels = { ‘No_Anomaly’ : A9, ‘Nosecone_Removed’: A1, ‘BothBodies_and_Nose_Removed’ : A2, ‘TopBody_and_Nose_Removed’ : A3, ,‘Body2_Removed’ : A4, ‘Door2_TimedOut’ : A5, ‘R04_crashed_nose’ : A6, ‘R03_crashed_tail’ : A7, ‘ESTOPPED’ : A8}

**Table A1:** Initial experiments on anomaly detection ^∗^.

AT	P	R	F1	S	A
A9	0.91	1	0.95	36,383	0.91
A1	0.75	0.06	0.1	1813	
A2	0.98	0.18	0.31	904	
A3	0.89	0.06	0.11	1145	
A4	0.96	0.63	0.76	636	
A5	1	1	1	792	
A6	1	0.99	1	332	
A7	1	0.95	0.97	255	
A8	0.61	0.98	0.75	49	
**MA**	0.9	0.65	0.66	42,309	

^∗^ AT—Anomaly type, P—Precision, R—Recall, F1—F1 Score, A—Accuracy, S—Support, MA—Macro average, A1:A9—see Table 3.

**Table A2:** Initial experiments on anomaly prediction ^∗^.

	P	R	F1	S	A
NA	0.9	0.9	0.9	3516	0.97
A	0.5	0.1	0.1	121	
**MA**	0.7	0.5	0.5		

^^∗^^ NA—Not Anomalous, A—Anomolous, P—Precision, R—Recall, F1—F1 Score, A—Accuracy, S—Support, MA—Macro average.

## Appendix D. More Details on TimeGPT Model

TimeGPT [16,54] is a pre-trained generative model for time series data developed by Nixtla. It is capable of generating precise predictions for untrained time series utilizing solely past values as inputs. Unlike traditional LLMs, TimeGPT focuses specifically on time series forecasting tasks such as demand forecasting, anomaly detection, and financial forecasting. In implementing the TimeGPT model, we followed these steps: tokenizing the description column, dividing the data into training and testing sets (97,510 and 2000 rows, respectively), and addressing TimeGPT’s forecasting limitations of only predicting 2000 values similar to the last 20–30 values of the training dataset. We structured the data to include a mix of non-anomalous (0) and anomalous (9) values in the final 20 entries. Both datasets were scaled using a standard scaler. Training data were then converted from a wide to a long format and were assigned a column name as a unique ID. Here, *unique_id* is the new column that indicates the original series, and *value* is the corresponding value for each series on each date. We renamed *_time* as *ds* and invoked the *timegpt.forecast* method using the *timegpt-1-long-horizon* model to predict 2000 values, and we used the *finetuning_steps* parameter to increase the accuracy. The final dataframe included *ds*, *Parameter 1*, and *Description*. Here, *Parameter 1* is the feature, and *Description* is the response variable of the dataset. Predicted values were adjusted to match the original data, and the RMSE and MSE scores were calculated for both *Description* and *Parameter 1*. A classification report was generated for the *Description* column, reflecting the model’s two-class predictions. Finally, we produced saved individual predictions with descaled *Parameter 1* values and used redefined predicted description values for the *Description* column.
